# Macrolide Antibiotics Exhibit Cytotoxic Effect under Amino Acid-Depleted Culture Condition by Blocking Autophagy Flux in Head and Neck Squamous Cell Carcinoma Cell Lines

**DOI:** 10.1371/journal.pone.0164529

**Published:** 2016-12-15

**Authors:** Kazuhiro Hirasawa, Shota Moriya, Kana Miyahara, Hiromi Kazama, Ayako Hirota, Jun Takemura, Akihisa Abe, Masato Inazu, Masaki Hiramoto, Kiyoaki Tsukahara, Keisuke Miyazawa

**Affiliations:** 1 Department of Otolaryngology (Head and Neck Surgery), Tokyo Medical University, Tokyo, Japan; 2 Department of Biochemistry, Tokyo Medical University, Tokyo, Japan; 3 Department of Breast Surgery, Tokyo Medical University, Tokyo, Japan; 4 Institute of Medical Science, Tokyo Medical University, Tokyo, Japan; University of Hong Kong, HONG KONG

## Abstract

Autophagy, a self-digestive system for cytoplasmic components, is required to maintain the amino acid pool for cellular homeostasis. We previously reported that the macrolide antibiotics azithromycin (AZM) and clarithromycin (CAM) have an inhibitory effect on autophagy flux, and they potently enhance the cytocidal effect of various anticancer reagents *in vitro*. This suggests that macrolide antibiotics can be used as an adjuvant for cancer chemotherapy. Since cancer cells require a larger metabolic demand than normal cells because of their exuberant growth, upregulated autophagy in tumor cells has now become the target for cancer therapy. In the present study, we examined whether macrolides exhibit cytotoxic effect under an amino acid-starving condition in head and neck squamous cancer cell lines such as CAL 27 and Detroit 562 as models of solid tumors with an upregulated autophagy in the central region owing to hypovascularity. AZM and CAM induced cell death under the amino acid-depleted (AAD) culture condition in these cell lines along with CHOP upregulation, although they showed no cytotoxicity under the complete culture medium. CHOP knockdown by siRNA in the CAL 27 cells significantly suppressed macrolide-induced cell death under the AAD culture condition. CHOP^-/-^ murine embryonic fibroblast (MEF) cell lines also attenuated AZM-induced cell death compared with CHOP^+/+^ MEF cell lines. Using a tet-off *atg5* MEF cell line, knockout of *atg5*, an essential gene for autophagy, also induced cell death and CHOP in the AAD culture medium but not in the complete culture medium. This suggest that macrolide-induced cell death via CHOP induction is dependent on autophagy inhibition. The cytotoxicity of macrolide with CHOP induction was completely cancelled by the addition of amino acids in the culture medium, indicating that the cytotoxicity is due to the insufficient amino acid pool. These data suggest the possibility of using macrolides for “tumor-starving therapy”.

## Introduction

Head and neck squamous cell carcinomas (HNSCCs) occur frequently with more than 500,000 new cases diagnosed worldwide each year [1]. Recurrent and/or metastatic HNSCC patients receive platinum-based chemotherapy as a first-line treatment [2]. As 80%-100% of HNSCCs have an increased expression level of epidermal growth factor receptor (EGFR), cetuximab, a humanized IgG monoclonal antibody against EGFR, has become increasingly used in combination therapy [3]. However, only few patients show long-term responses, indicating that more effective therapeutic strategies are required for improved therapeutic outcome [4].

Cancer cells grow faster than normal cells *in vivo*, and they are believed to exhibit a higher metabolic demand [5]. In particular, HNSCC cells with increased activation of EGFR-related signals require a larger metabolic demand. To meet the amino acid demand, cancer cells increase the expression level of amino acid transporters such as LAT1, as well as the activation of autophagy for degradation of their cytoplasmic proteins and organelles to recycle the intracellular amino acids [6, 7]. Autophagy is a self-eating process wherein cells capsulate their own cytoplasmic proteins and organelles in double-membrane vesicles to form autophagosomes. They are transported to the lysosome following membrane fusion to each other (autolysosomes). The contents of autolysosomes are finally degraded by lysosomal hydrolases [8]. The resulting breakdown products are used as a source of energy and new protein synthesis [9]. Autophagy is indispensable for cell survival and is enhanced under a nutrient-starved condition, particularly in solid tumors because of their excessive metabolic requirements and hypovascular condition [10, 11].

We previously reported that clinically well-used macrolide antibiotics, such as azithromycin (AZM) and clarithromycin (CAM), block autophagy flux [12, 13]. In multiple myeloma and breast cancer cells, we found that apoptosis induction was enhanced by the proteasome inhibitor bortezomib and AZM or CAM combination treatment together with endoplasmic reticulum (ER)-stress loading [12, 14]. In terms of drug-repositioning, AZM and CAM are considered strong candidates as clinical autophagy inhibitors. There is, however, still a need to further clarify the specific molecular mechanism responsible for macrolide-induced autophagy inhibition [15].

In the current study, we first show that AZM and CAM exert a potent cytotoxic effect on HNSCC cell lines only under amino acid-depleted (AAD) culture conditions. We further discuss the possibility of “tumor-starving therapy” using macrolide antibiotics in HNSCC patients.

## Materials and Methods

### Reagents

AZM was purchased from Tokyo Chemical Industry (Tokyo, Japan), and was dissolved in dimethyl sulfoxide (DMSO) to prepare 50 mM stock solutions. CAM was purchased from Wako Pure Chemical Industries (Osaka, Japan) and was dissolved in ethanol to prepare 5 mM stock solutions. E-64d and pepstatin A, which are inhibitors of lysosomal proteases, and Z-VAD-fmk, which is a pan-caspase inhibitor, were all purchased from Peptide Institute (Osaka, Japan). Necrostatin-1, a specific inhibitor of RIPK1, was purchased from Enzo Life Sciences (Farmingdale, NY, USA). Thapsigargin was purchased from Nacalai Tesque (Kyoto, Japan). Doxycycline hydrochloride, Chloroquine diphosphate, DL-α-Tocopherol and Astaxanthin were from Sigma-Aldrich (St. Louis, MO, USA). Amino acid-free Dulbecco’s Modified Eagle’s Medium (DMEM) (048–33575), MEM Essential Amino Acids Solution (100x) (132–15641) and MEM Non-essential Amino Acids Solution (50x) (139–15651) were purchased from Wako Pure Chemical Industries.

### Cell lines and culture conditions

The human oral squamous cell carcinoma cell line CAL 27 and the human pharyngeal carcinoma cell line Detroit 562 obtained from the American Type Culture Collection (ATCC, Manassas, VA, USA) were cultured in DMEM plus 10% fetal bovine serum (FBS) (Biowest, Nuaillé, France), and 1% penicillin/streptomycin solution (Wako Pure Chemical Industries). A CHOP^-/-^ murine embryonic fibroblast (MEF) cell line (CHOP-KO-DR) established from a 13.5-day-old CHOP^-/-^ mouse embryo by SV-40 immortalization and a CHOP^+/+^ MEF cell line (DR-wild-type) established by SV-40 immortalization as a control cell line for CHOP-KO-DR were also obtained from the ATCC, and were cultured in DMEM plus 10% FBS and 1% penicillin and streptomycin. The “AAD culture medium” described in this manuscript indicates amino acid-free DMEM plus 10% FBS and 1% penicillin and streptomycin. Normal DMEM contains a sufficient amount of 20 amino acids including 75 mg/L L-glutamic acid and 30 mg/L L-methionine, whereas amino acid-free DMEM contains no amino acids; AAD culture medium (amino acid-free DMEM plus 10% FBS) only contains amino acids less than 1/10 of complete DMEM with 10% FBS. The m5-7 cell line, an *atg5* tet-off MEF system, was a kind gift from Dr. Noboru Mizushima (The University of Tokyo, Tokyo, Japan). Details of the culture conditions for passage and the condition for knock-out of the *atg5* gene for complete autophagy inhibition were previously described [16]. All cell lines were cultured in a humidified incubator containing 5% CO2 and 95% air at 37°C. All cell lines were used for the experiments within 5 passages after thawing.

### Assessment of cell growth inhibition and apoptosis induction

Cell growth inhibition was measured by the Cell Titer-Blue cell viability assay (Promega, Madison, WI, USA). Cells were treated with or without drugs for 24, 48, and 72 hrs in 96-well plates. In the last 4 hrs, the Cell Titer-Blue reagent was added to each well, and fluorescence was measured at 560 nm excitation and 590 nm emission. The percentage of the mean fluorescence measured to that in untreated cells was expressed as % cell growth inhibition.

For assessment of apoptosis, cells were stained with Annexin V and propidium iodide (PI) using APOPCYTOTM Annexin V-Azami-Green Apoptosis Detection kit (MBL, code 4690, Nagoya, Japan) according to the manufacturer's instructions and subjected to flow cytometry using Attune^®^ Acoustic Focusing Cytometer (Life Technologies, CA, USA).

### Immunoblotting

Immunoblotting was performed as previously described in detail [17]. Briefly, cells were lysed with RIPA lysis buffer (Nacalai Tesque) containing 1 mM PMSF, 0.15 U/ml aprotinin, 10 mM EDTA, 10 ng/ml sodium fluoride and 2 mM sodium orthovanadate. Cellular proteins were quantified using a DC Protein Assay (Bio-Rad, Richmond, CA). Equal amounts of proteins were loaded onto the gels, separated by SDS-PAGE and transferred to an Immobilon-P membrane (Millipore, Bedford, MA, USA). The membranes were probed with primary antibodies (Abs) such as anti-microtubule- associated protein 1 light chain 3 (LC3) B antibody (Ab) (NB600-1384; Novus Biologicals, Inc., Littleton, CO), and anti-phosoho-eIF2α (Ser51) Ab (#9721S), anti-CHOP (GADD153) monoclonal (m) Ab (#2895S), anti-p70S6K Ab (#9202S), anti-phospho-p70S6K (Thr389) Ab (#9205S), anti-PARP Ab (#9542) (Cell Signaling Technology, Danvers, MA, USA), and anti-p62 (sequestosome-1) mAb (sc-28359), anti-GAPDH mAb (sc-32233), and anti-β-actin mAb (sc-47778) (Santa Cruz Biotechnology, Santa Cruz, CA, USA) and anti-XBP-1s Ab (S647501) (BioLegend, San Diego, CA). Immunoreactive proteins were detected with horseradish peroxidase-conjugated second Abs (Jackson, West Grove, PA) and an enhanced chemiluminescence reagent (ECL) (Millipore). Densitometry was performed using a Molecular Imager, ChemiDoc XRS system (Bio-Rad).

### Detection of intracellular reactive oxygen species

Reactive oxygen species (ROS) was quantified using ROS-Glo^™^ H_2_O_2_ Assay (Promega) according to the manufacture’s instruction. Cells were treated on a white 96-well plate with the medium plus the test compound (total: 80 μL). To each well, 20 μL of H_2_O_2_ substrate solution was added, and cells were incubated at 37°C/5% CO_2_ for 6 hrs. After incubation, 100 μL of the detection solution was added. After 20 min incubation at room temperature, the relative luminescence was measured.

### Transfection of CHOP siRNAs

For the gene silence of CHOP in CAL 27 cells, CHOP siRNA (VHS40605) and a control siRNA (12935–300), whose sequences are described below, were purchased from Thermo Fisher Scientific., and diluted to a final concentration of 33 nM in Opti-MEM I (Thermo Fisher Scientific). Transfection was performed with the cells at 50% confluency using Lipofectamine RNAi MAX transfection reagent (Thermo Fisher Scientific) according to the manufacturer’s instruction.

CHOP sense: UUUCCUGCUUGAGCCGUUCAUUCUC, CHOP antisense: GAGAAUGAACGGCUCAAGCAGGAAA, Control sense and antisense: not shown

### Assessment of morphology in cultured cells

Cells were spread on slide glasses using Cytospin 4 Centrifuge (Thermo Fisher Scientific, Inc., Rockford, IL, USA) to make slide glass preparations. Preparations were then stained with May-Grünwald-Giemsa, and examined using a degital microscope BZ-8000 (Keyence Co., Osaka, Japan).

### Statistical analysis

All quantitative data were expressed as mean ± standard deviation (SD). Statistical analysis was performed using two-tailed nonpaired Student’s t-test. The criterion for statistical significance was taken as p < 0.05.

## Results

### AZM and CAM block autophagy flux and induce cell death under the amino acid-depleted culture condition

**Fig 1:**
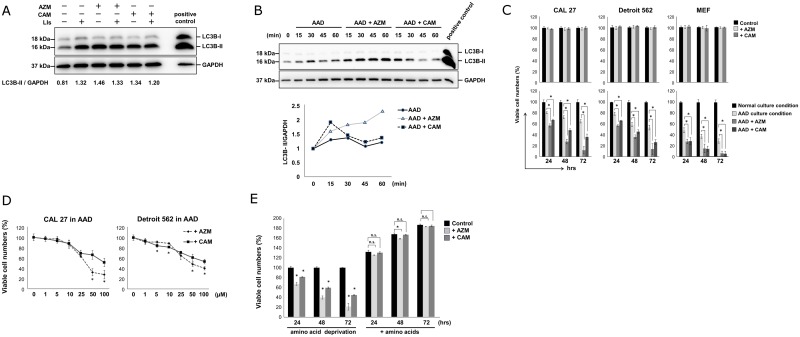
Macrolides induce cell death in amino acid-depleted culture by blocking autophagy flux. (A) Immunoblotting with LC3B and p62 Abs from the cell lysates of CAL 27 cells cultured in the complete culture medium with/without AZM, CAM (50 μM) in the presence or absence of lysosomal inhibitors (LIs) E-64d (10 μg/mL) and pepstatin A (10 μg/mL) for 24 hrs. Immunoblotting with anti-GAPDH mAb was used as an internal control. Cell lysate derived from PANC-1 cells treated with AZM for 24 hrs was used as a positive control [13]. (B) (upper panel) Immunoblotting of LC3 from the cell lysates of CAL 27 cells cultured in the complete culture medium or AAD culture medium containing 10% FBS with or without AZM (50 μM) and CAM (50 μM) for up to 60 min. Immunoblotting with anti-GAPDH mAb was used as an internal control. Cell lysate derived from PANC-1 cells treated with AZM (50 μM) for 24 hrs was used as a positive control [13]. (lower panel) The ratios of LC3B-II/GAPDH were plotted. (C) Cell growth inhibition of CAL 27, Detroit 562, and MEF cells treated with AZM (50 μM) and CAM (50 μM) in the complete culture medium (upper panel) and AAD culture medium (lower panel) for 24, 48, and 72 hrs. Viable cell numbers are expressed as percentage to viable cells cultured in the complete culture medium at each indicated culture period. Data are presented as means ± SEM. *p < 0.05. (D) CAL 27, Detroit 562, and MEF cells were cultured under the AAD culture condition in the presence of AZM or CAM at various concentrations for 48 hrs. Data are presented as means ± SEM. *p < 0.05; AZM vs CAM. (E) Effects of supplementation of amino acids to the AAD culture medium on CAL 27 cells under the AAD culture condition with or without macrolides. ‘Amino acids’ indicate 1% MEM non-essential amino acids plus 2% MEM essential amino acids at the final concentration (Wako). The numbers of viable cells were determined and the results were expressed as the percentage against the numbers of viable cells cultured under the AAD culture condition without macrolides at various time points, namely, 24-hr, 48-hr, and 72-hr cultures. *p < 0.05 (vs without macrolides). ‘n.s.’ indicates ‘not significant’.

We initially confirmed that AZM and CAM inhibit autophagy in HNSCC cell lines as previously described in myeloma and breast cancer cell lines by flux analysis [12, 14, 18]. The conversion from the soluble cytosolic LC3B-I with a molecular weight of 16 kDa to the membrane-bound LC3B-II with a molecular weight of 18 kDa via the conjugation of phosphatidyl ethanolamine represents the formation of autophagosomes. Therefore, LC3B-II expression is a good marker for autophagosomes [18]. We cultured CAL 27 cells with macrolides in the presence or absence of lysosomal inhibitors (E-64d and pepstatin A) for 24 hrs in the complete culture medium, and performed immunoblotting with anti-LC3B Ab. As shown in Fig 1A, AZM or CAM treatment upregulated the LC3B-II expression. However, in the presence of the lysosomal inhibitors, the LC3B-II expression did not further increase compared with CAL 27 cells treated with macrolide or lysosomal inhibitors alone. This indicates that both AZM and CAM block autophagy flux in CAL 27 cells. Thereafter, we examined autophagy induction under the AAD culture condition in the presence or absence of macrolides by tracing LC3B-II expression (Fig 1B). CAL 27 cells were washed twice with PBS and cultured in AAD Dulbecco’s Modified Eagle’s Medium (DMEM) containing 10% FBS. After amino acid depletion, the expression ratios of LC3B-II to GAPDH augmented within 15 min and further increased at 30 min, but attenuated at 45 min. In the presence of AZM or CAM, the ratios of LC3B-II/GAPDH further increased at 15 min compared with the CAL 27 cells cultured in the AAD culture medium. This appears to be due to the accumulation of autophagosomes by autophagy induction in response to amino acid starvation as well as the inhibition of autophagosome degradation by macrolides simultaneously in the autophagy flux. Worth noting was that the LC3B-II upregulation by CAM was transient, whereas the LC3B-II upregulation by AZM persisted and further increased during 60 min exposure. This may be because AZM is more potent than CAM in blocking autophagy flux as we previously described in pancreatic cell lines; however, other molecular mechanisms may be involved [13].

We next examined the cell growth inhibition of CAL 27 and Detroit 562 cells under the AAD culture condition. The AAD culture medium showed some cell growth inhibition compared with the complete culture medium (Fig 1C). Although the addition of AZM and CAM at 50 showed no cytotoxicity in the complete culture medium, these macrolide antibiotics induced marked reduction in the viable cell number in the AAD culture medium in both cell lines as well as in MEF cells in a time- and dose-dependent manner (Fig 1C and 1D). In addition, treatment of CAL 27 cells with chloroquine, a well-known autophagy inhibitor [18], markedly decreased the viable cell number in the AAD culture medium (S1 Fig). The cytotoxicity of AZM was more potent than that of CAM in HNSCC cell lines, and there appeared to be a good correlation with the efficacy for blocking autophagy shown in Fig 1B. Therefore, blocking the efficiency of autophagy appeared to be involved in the mechanism of cytotoxicity under the AAD culture condition. Notably, the addition of amino acids in the AAD medium completely cancelled the cytotoxic effect of macrolides in CAL 27 cells (Fig 1E). When we added the essential and non-essential amino acids respectively in the AAD medium, supplementation of the essential amino acids has more efficiently but not completely cancelled the AZM-induced cell death than supplementation of the non-essential amino acids (S2 Fig). This indicates that the cytotoxic effect of macrolides can be induced only under the shortage of intracellular amino acids.

**Fig 2:**
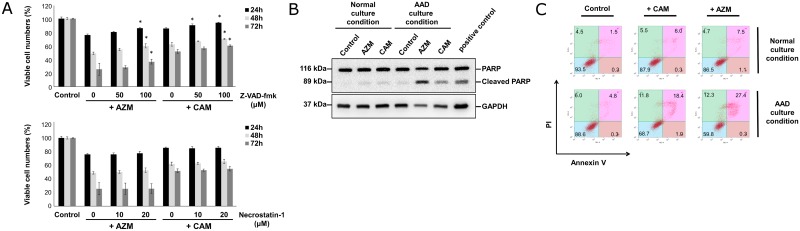
Apoptosis induction after treatment with macrolides under AAD culture condition. (A) CAL 27 cells in the AAD culture medium containing 10% FBS with or without macrolides were treated with various concentrations of either Z-VAD-fmk (upper panel) or necrostatin-1 (lower panel). Data are presented as means ± SEM. *p < 0.05 (vs without Z-VAD-fmk/necrostatin-1). (B) Immunoblotting with PARP Ab from the cell lysates of CAL 27 cells cultured in the complete culture medium or AAD culture medium with or without macrolides for 12 hrs. Immunoblotting with anti-GAPDH mAb was used as an internal control. Cell lysate derived from HL-60 cells treated with vitamin K2 for 48 hrs was used as a positive control for apoptosis induction [17]. (C) Flow cytometry with annexin V/PI double staining after 24-hr treatment of CAL 27 cells with AZM/CAM (50 μM) in the complete culture medium or AAD culture medium containing 10% FBS. Numbers indicate the percentage of the cells in each area.

To examine whether apoptosis is involved in the cytotoxicity of macrolides, CAL 27 cells were treated with AZM/CAM in the AAD culture medium in the presence or absence of a pan-caspase inhibitor, Z-VAD-fmk, or an RIP1 kinase inhibitor, necrostatin-1 [13]. As shown in Fig 2A, Z-VAD-fmk, but not necrostatin-1, attenuated the macrolide-induced cytotoxicity in a dose-dependent manner. In addition, immunoblotting with anti-PARP Ab showed the cleavage of PARP in AZM/CAM-treated CAL 27 cells under the AAD culture condition (Fig 2B). Furthermore, flow cytometry showed an increase in the number of Annexin V/PI-positive cells (Fig 2C). All these data indicate apoptosis induction in response to AZM/CAM treatment under the AAD culture condition.

**Fig 3:**
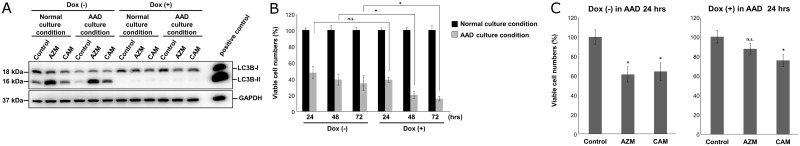
Effects of autophagy inhibition on macrolide-induced cytotoxicity in tet-off *atg5* MEF cell line. (A) m5-7 cells were pre-treated with/without doxycycline (Dox, 10 ng/mL) for 7 days for complete autophagy inhibition. Subsequently, the cells were further treated with/without macrolides for 24 hrs under the complete culture or AAD culture conditions. The cellular proteins were separated by 15% SDS-PAGE, and immunoblotted with LC3B Ab. Immunoblotting with anti-GAPDH mAb was used as an internal control. The positive control was the cell lysate derived from PANC-1 cells treated with AZM (50 μM) for 24 hrs and used in a previous report [13]. (B) After Dox treatment, m5-7 cells were cultured in the complete culture medium or AAD culture medium containing 10% FBS for 24 hrs. Viable cell numbers were assessed and expressed as percentage to the viable cells cultured in the complete culture medium at each indicated culture period. Data are presented as means ± SEM. *p < 0.05 (Dox (-) vs Dox (+)). (C) Cell growth inhibition of m5-7 cells cultured under the AAD culture condition with or without macrolides for 24 hrs. Data are presented as means ± SEM. *p < 0.05 (vs control). ‘n.s.’ indicates ‘not significant’.

We next confirmed the dependency on autophagy inhibition for macrolide-induced cell death under the AAD culture condition using the m5-7 cell line, which is the *atg5* tet-off MEF system [16]. Pretreatment of m5-7 cells with doxycycline (Dox) for knockout of the *atg5* gene resulted in complete autophagy inhibition as exhibited by the absence of LC3B-II induction in any of the conditions tested (Fig 3A). Under the AAD culture condition, Dox-pretreated m5-7 cells apparently showed a more reduced cell viability than Dox-untreated control cells (Fig 3B). Under complete autophagy inhibition in Dox (+) m5-7 cells, AZM treatment did not induce further enhancement of cell growth inhibition (Fig 3C). This indicates that the cytotoxicity of macrolides is directly linked with autophagy inhibition.

### Involvement of CHOP induction for macrolide-induced cell death under AAD culture condition

**Fig 4:**
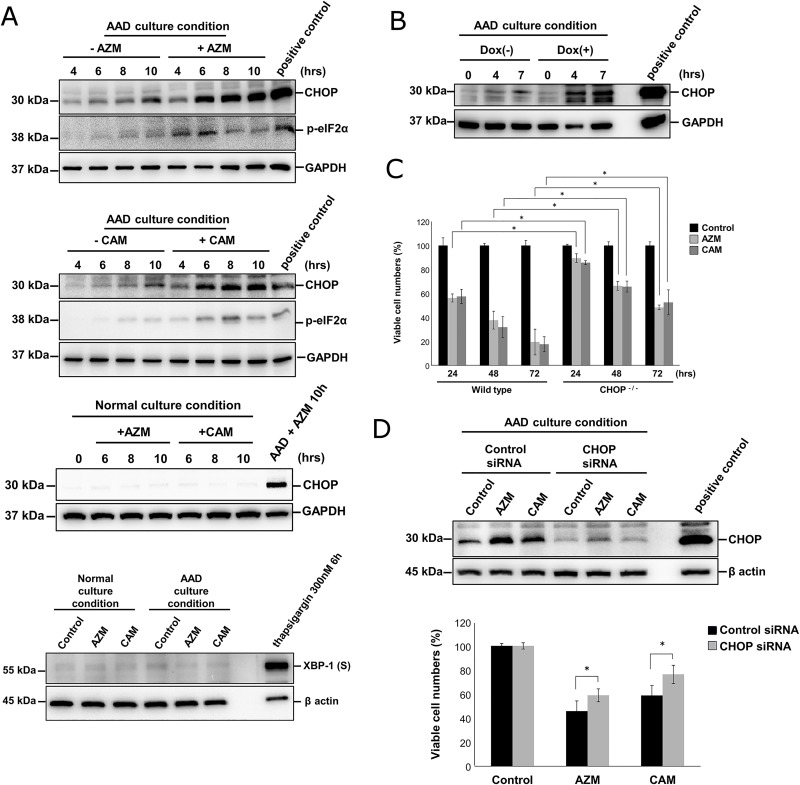
Macrolides induce cell death of CAL 27 cells under AAD culture condition via CHOP induction. (A) (1) CAL 27 cells were cultured in the AAD culture medium containing 10% FBS with/without macrolides for various time lengths. Cellular proteins were separated by 11.25% SDS-PAGE and immunoblotted with CHOP and phospho-eIF2α Abs. The positive control was the CAL 27 cell lysates cultured in the complete culture medium with thapsigargin (300 nM) for 6 hrs. Immunoblotting with anti-β-actin Ab was used as an internal control. (2) Immunoblotting was performed with CHOP mAb from the cell lysates of CAL 27 cells cultured in the complete culture medium with macrolides for the indicated time periods. The same cell lysate of CAL 27 cells cultured in the AAD culture medium with AZM (50 μM) for 10 hrs was also loaded on the gel. Immunoblotting with anti-GAPDH mAb was used as an internal control. (3) Immunoblotting was performed with XBP-1s Ab from the cell lysates of CAL 27 cells cultured in the AAD culture medium containing 10% FBS with macrolides for 8 hrs. The positive control was the CAL 27 cell lysates cultured in the complete culture medium with thapsigargin (300 nM) for 6 hrs. Immunoblotting with anti-β-actin mAb was used as an internal control. (B) m5-7 cells with/without pre-treatment with Dox were further cultured with macrolides in the AAD culture medium containing 10% FBS for 24 hrs. Immunoblotting was performed using anti-CHOP mAb. The positive control was the CAL 27 cell lysates cultured in the complete culture medium with thapsigargin (300 nM) for 6 hrs. Immunoblotting with anti-GAPDH mAb was used as an internal control. (C) Wild-type MEF cells and CHOP ^-/-^ MEF cells were cultured in the AAD culture medium containing 10% FBS with/without macrolides. Data are presented as means ± SEM. *p < 0.05. (D) Effects of CHOP knockdown by siRNA on CAL 27 cells: (upper panel) The expression levels of CHOP were analyzed by immunoblotting using lysates of CAL 27 cells to which either control siRNA or CHOP siRNA was introduced and cultured under the AAD culture condition with or without macrolides for 8 hrs. The positive control was the CAL 27 cell lysates cultured in the complete culture medium with thapsigargin (300 nM) for 6 hrs. After 8 hrs of treatment with control siRNA and CHOP siRNA, the CAL 27 cells were further cultured under the AAD culture condition in the presence/absence of macrolides for 48 hrs, and viable cell numbers were assessed. Data are presented as means ± SEM. *p < 0.05.

It was reported that eIF2α is phosphorylated via GCN2 activation in response to nutrient deprivation, following the induction of a pro-apoptotic transcriptional factor CHOP/GADD153 [19, 20]. Therefore, we speculated that CHOP might be involved in our system. As shown in Fig 4A-1, CHOP was induced in a time-dependent manner under the AAD culture condition, and its expression became more pronounced in the presence of AZM/CAM (Fig 4A-1). Along with CHOP induction, eIF2α was phosphorylated to some extent, but attenuated at more than 8 hrs longer exposure to AZM under the AAD culture condition. In contrast to the AAD culture condition, we observed no CHOP induction by AZM/CAM in the cells cultured in the complete culture medium (Fig 4A-2).

Although the treatment of CAL 27 cells with thapsigargin, a typical ER-stress inducer via disturbance of the calcium homeostasis, resulted in the induction of XBP-1, macrolide treatment exhibited no XBP-1s induction under the AAD culture condition (Fig 4A-3). Thus, CHOP induction appears to be independent from the canonical UPR pathways [21]. Furthermore, in atg5 tet-off m5-7 cells, knockout of the *atg5 gene* resulted in pronounced CHOP induction under the AAD culture condition (Fig 4B). This indicates that autophagy inhibition enhances CHOP induction under the AAD culture condition.

To investigate whether CHOP is involved in cell death induction, we used a CHOP knockout MEF cell line. Under the AAD culture condition, CHOP ^-/-^ MEF cells maintained their considerably higher viability than wild-type MEF cells in the presence of AZM/CAM in the AAD culture medium (Fig 4C). Furthermore, we introduced CHOP siRNA into the CAL 27 cells. Knockdown of CHOP significantly attenuated the cytotoxic effects of AZM and CAM (Fig 4D). Therefore, macrolide-induced CHOP appears to be involved in cell death induction.

**Fig 5:**
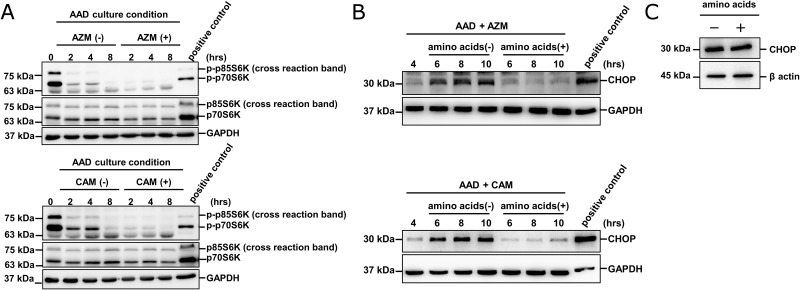
CHOP induction by macrolides under AAD culture condition in accordance with intracellular amino acid starvation in CAL 27 cells. (A) After treatment of CAL 27 cells with/without macrolides in the AAD culture medium containing 10% FBS for various lengths of time, the cells were lysed and cellular proteins were separated by 11.25% SDS-PAGE and immunoblotting with anti-phosphor-p70S6K (Thr389) and anti-p70S6K Abs. The positive control was the cell lysate derived from Detroit 562 cells having constitutively activated PI3K mutation. Immunoblotting with anti-GAPDH mAb was used as an internal control. (B) Addition of amino acids into the culture medium canceled CHOP induction: After 4 hrs of depletion of amino acids in the culture medium in the presence of AZM/CAM (50 μM), amino acids were added at final concentrations of 1% MEM non-essential amino acids and 2% MEM essential amino acids, and subsequently cultured for the indicated time periods. Thereafter, cellular proteins were separated by 11.25% SDS-PAGE and immunoblotting with anti-CHOP mAb. The positive control was the CAL 27 cell lysates cultured in the complete culture medium with thapsigargin (300 nM) for 6 hrs. Immunoblotting with anti-GAPDH mAb was used as an internal control. (C) CAL 27 cells were cultured with thapsigargin (300 nM) for 6 hrs with/without the addition of amino acids into the cell culture medium as described above, and immunoblotted with anti-CHOP mAb. Immunoblotting with β-actin mAb was used as an internal control.

To examine whether the insufficiency of the amino acid pool is directly linked to the CHOP induction in our system, we focused on p70S6K, which is a substrate of mammalian target of rapamycin (mTOR). mTOR senses and responds to amino acid availability to modulate protein synthesis via the phosphorylation of threonine 389 in p70S6K [22]. To evaluate intracellular amino acid sufficiency, we assessed the phosphorylation of p70S6K using the specific phospho- p70S6K (Thr389) Ab, as previously reported [23] (Fig 5A). Under the AAD culture condition, phosphorylation of p70S6K was attenuated. When AZM or CAM was added in the AAD culture medium, the suppression of the p70S6K phosphorylation was further pronounced. These results suggest that the amino acid pool was satisfied by autophagy in the absence of macrolides, whereas the amino acid pool became exhausted in the presence of macrolides. Furthermore, the addition of amino acids in the AAD culture medium completely abolished CHOP induction (Fig 5B). As thapsigargin treatment induced CHOP regardless of the addition of amino acids (Fig 5C), the inhibition of CHOP induction shown in Fig 5B is not due to the direct transcriptional effect of amino acids, but is a result of insufficiency of the amino acid pool.

## Discussion

**Fig 6:**
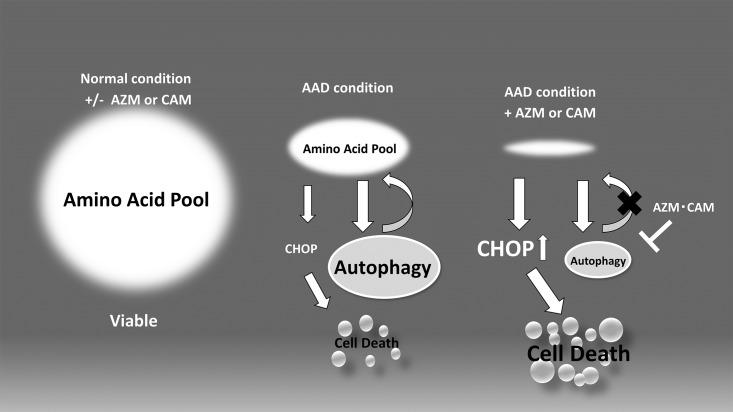
Proposed scheme for cell death induction by macrolide antibiotics under amino acid-depleted culture condition. Under the amino acid-depleted culture condition, autophagy is induced as an adaptive response. AZM/CAM block autophagy flux leading to the shortage of the intracellular amino acid pool and failure of cellular amino acid homeostasis. This results in the induction of an ER-stress-related pro-apoptotic transcription factor, CHOP.

In the present study, we showed that AZM and CAM blocked autophagy flux and induced cell death in HNSCC cells via CHOP induction when the cells were cultured in the AAD culture medium. Thus, we have drawn up a scheme of our observations as shown in Fig 6. In the complete culture medium, AZM and CAM showed no cytotoxicity in HNSCC cells because the amino acid pool was sufficient and CHOP was not upregulated (Figs 1C and 4A-2). Under the AAD culture condition, autophagy was induced to compensate the amino acid pool by recycling through a self-digestive mechanism. In this case, a pro-apoptotic transcription factor CHOP is induced at a lower level, which resulted in some reduction of the viable cell number (Figs 1C and 4A-1). However, in the presence of macrolide antibiotics under the AAD culture condition, the shortage of the amino acid pool cannot be augmented by autophagy because of the blocking effect on autophagy by the macrolides. This results in pronounced CHOP induction leading to enhanced cell death (Figs 1C and 4A-1).

CHOP is a well-known pro-apoptotic transcription factor induced in response to ER stress and amino acid deprivation [24, 25]. Under the AAD culture condition, the addition of AZM and CAM prevented the phosphorylation of threonine 389 in p70S6K (Fig 5A), which indicates the failure of amino acid supply by blocking autophagy. The addition of amino acids in the AAD culture medium completely abolished CHOP induction (Fig 5B). Therefore, CHOP was induced as a result of amino acid pool insufficiency. Since CHOP^-/-^ MEF attenuated the cytocidal effects of macrolides under the AAD culture condition compared with wild-type MEF, the transcriptional activation of CHOP is at least partly involved in the macrolide-induced cell death (Fig 4C). However, since CHOP^-/-^ MEF still exhibited some cytocidal effects in response to macrolides (Fig 4C), other mechanisms may be involved in the cytocidal effect of AZM/CAM under the AAD culture condition. It has been reported that nutrient starvation stimulates ROS production including H_2_O_2_ [26], and that the ROS accumulation induces autophagy [27]. It has also been reported that autophagy is induced to reduce oxidative stress via the clearance of damaged mitochondria (designated as ‘mitophagy’) and oxidized proteins [28, 29]. Therefore, we speculated that ROS might be accumulated via autophagy inhibition by AZM/CAM under the AAD culture condition, which may directly induce cell death. However, the addition of antioxidant reagents such as α-tocopherol and astaxanthin in the AAD culture medium did not prevent the cytotoxic effect of AZM/CAM on CAL 27 cells (S3A Fig). Furthermore, assessment of oxidative stress by ROS-Glo^™^ H_2_O_2_ assay revealed a higher oxidative stress loading in the cells cultured in the AAD culture condition regardless of the presence or absence of macrolides (S3B Fig). Thus, ROS accumulation does not appear to explain the enhanced cell death by macrolides.

In our system, CHOP induction appears to be due to the failure of amino acid homeostasis, as previously reported [23]. However, other mechanisms for CHOP induction have been reported such as UPR and ROS accumulation [21, 30]. If the AZM/CAM treatment induced accumulation of unfolded proteins by blocking the autophagy flux, these proteins should have induced more CHOP under the complete culture condition because of the larger source for *de novo* protein synthesis (i.e., amino acids) under the complete culture condition (Fig 4A-2). Furthermore, the spliced form of XBP1, which is known to be effectively induced by UPR [31], was not induced by macrolides under the AAD culture condition (Fig 4A-3). Additionally, as described above, although the AAD cell culture condition induced ROS production, macrolides did not further enhance ROS accumulation (S3B Fig). Thus, amino acid insufficiency appears to be the main axis for CHOP induction in our system.

We previously reported that AZM was more effective than CAM for blocking autophagy flux at the same concentration in the pancreatic cancer cell line PANC-1 [13]. In the CAL 27 and Detroit 562 cells, AZM exhibited a stronger cytotoxicity under the AAD culture condition than CAM (Fig 1B), which might be correlated with their blocking efficiency of autophagy. However, unlike AZM, CAM reduced the viable cell number in *atg5* knocked-out m5-7 cells which have no ability for autophagosome formation (Fig 3C). Thus, other molecular mechanisms might underlie the cytotoxic effect of CAM. In previous *in vivo* studies, CAM was reported to inhibit angiogenesis and TGF-iogenesisc e [32, 33]. To the best of our knowledge, there appears to be no report regarding the cytotoxic effect of CAM alone on cultured cells.

Our present results indicate that the cytotoxic effect of macrolides under the amino acid-starving condition appears to be due to at least partially apoptosis induction: cleavage of PARP and increased number of annexin V-positive cells. However, in microscopy, we could not observe the apparent apoptotic features in the CAL 27 cells treated with AZM under the AAD culture condition, such as formation of apoptotic bodies, chromatin condensation, and nuclear fragmentation, although the cells showed many intercellular vacuoles (S4 Fig). This might be the typical features of type II programmed cell death called ‘autophagic cell death’ [34]. However, in tet-off-*atg5* m5-7 cells, blocking autophagy did not rescue the macrolide-induced cell death (S5 Fig). This indicates that autophagic cell death is not involved in our system. Recently, other types of cell death with vacuolization called paraptosis [35, 36], oncosis [37], and methuosis [38] have been reported, although these types of cell death should show a slight response or no response to caspase inhibitors, unlike our result shown in Fig 2A [38]. These lines of evidence suggest that the CHOP axis in response to amino acid deprivation plays an important role for the macrolide-induced cytotoxic effect, although there might be more than one molecular mechanism as well as type of cell death.

AZM and CAM block autophagy flux as shown in Fig 1A. In the clinical setting, it has been reported that the long-term use of AZM in patients with cystic fibrosis increased nontuberculous mycobacteria infection owing to autophagy inhibition by AZM [39]. However, the molecular mechanism of blocking autophagy flux by AZM and CAM is not well understood. It is reported that macrolide antibiotics interact with valosin-containing proteins (VCPs) without mediating their anti-inflammatory activities [40], and that VCPs are essential for the maturation of autophagosomes [41, 42]. However, transfection of various VCP variants including the AAA-ATPase-deficient form into HEK293T cells did not show any effect on AZM-induced autophagy (data not shown). Therefore, VCPs do not appear to be involved in the macrolide-induced autophagy inhibition. The selective inhibition of a proton-pumping V-ATPase by bafilomycin A1 (BAF), a macrolide and a well-known in vitro autophagy inhibitor, was previously initially evaluated [43]. BAF induces vesicular proton gradient disruption and pH increase in acidic vesicles (e.g., lysosomes). The vesicular acidification disruption by BAF possibly prevents the fusion of autophagosomes with lysosomes, causing autophagy inhibition. The reported increase in macrophage lysosomal pH by AZM may result in inhibition of lysosomal hydrolases with an optimal low pH for their enzymatic activities [39]. According to these reports, AZM and CAM possibly disrupt vesicular acidification and inhibit the fusion of autophagosomes with lysosomes, resulting in the blockage of autophagy flux similarly to BAF. We are currently investigating the molecular target(s) of AZM/CAM for blocking autophagy.

To date, several drugs targeting tumor angiogenesis, such as the VEGF inhibitor bevacizumab, have been used in the clinical setting [44]. These drugs are believed to starve tumor cells by ischemia via the induction of hypovascularization, leading to the upregulation of autophagy in the central region of solid tumors [45, 46]. Thus, blocking autophagy with macrolides may effectively enhance the cytotoxic effect of VEGF inhibitors. In fact, it has been reported that blocking autophagy with chloroquine enhanced the cytotoxicity of bevacizumab in hepatocarcinoma, although the report suggested that the synergistic anticancer effect was due to ROS generation [47]. Recently, amino acid transporters have been extensively investigated in cancer cells. It has been reported that cancer cells highly express an L-type amino acid transporter 1 (LAT1), whereas normal cells highly express LAT2 [48, 49]. At present, an LAT1-specific inhibitor is undergoing phase I clinical trial. Therefore, macrolides could be potentially used by the combination of these drugs in cancer patients.

## Conclusions

We demonstrated that AZM and CAM block autophagy flux and cause cell death under the AAD culture condition via CHOP induction owing to the failure of internal amino acid homeostasis. This study indicates the possibility of using macrolides as a novel ‘tumor-starving therapy’ in cancer treatment.

## Supporting Information

S1 Fig Cell growth inhibition of CAL 27 cells under normal or AAD culture condition in the presence/absence of chloroquine. CAL 27 cells were cultured with/without 50 μM chloroquine either in the complete culture medium or AAD culture medium for the indicated period of time. Then, viable cell number was assessed and expressed as percentage to the viable cells cultured in the complete culture medium at each indicated culture period. Data are presented as means ± SEM. *p < 0.05.
(TIF)

S2 Fig Effects of supplementation of essential and non-essential amino acids in AZM-induced cytotoxicity under the AAD culture condition on CAL 27 cells. CAL 27 cells were cultured in the AAD culture medium supplemented with essential and non-essential amino acids with/without AZM (50 μM). ‘Essential’ and ‘non-essential’ indicate 2% MEM essential amino acids and 1% MEM non-essential amino acids at the final concentration (Wako), respectively. The number of viable cells was determined and compared with that of viable cells cultured under the AAD culture condition without AZM. *p < 0.05. ‘n.s.’ indicates ‘not significant’.
(TIF)

S3 Fig Effects of ROS in macrolide-induced cytotoxicity on CAL 27 cells. (A) CAL 27 cells were cultured with macrolides under the AAD culture condition with 10% FBS with/without the two types of ROS scavengers, namely, α-tocopherol (50 μM) and astaxanthin acid (25 μM) for 48 hrs. (B) CAL 27 cells were cultured with macrolides under the complete or AAD culture condition for 6 hrs. ROS production was assessed using ROS-Glo^™^ H_2_O_2_ Assay (Promega) as described in Materials and Methods. ‘n.s.’ indicates ‘not significant’.
(TIF)

S4 Fig Morphological changes after macrolide treatment in CAL 27 cells. May-Grünwald-Giemsa staining was performed after treatment with or without macrolides under the normal or AAD culture condition for 24 hrs.
(TIF)

S5 Fig Effects of autophagy inhibition on macrolide-induced cytotoxicity under AAD culture condition. m5-7 cells with/without pretreatment with Dox (10 ng/mL) were cultured under the normal culture or AAD culture condition with AZM/CAM (50 μM) for 24 hrs. Viable cell number is expressed as the percentage of viable m5-7 cells with/without Dox under the normal culture condition. Data are presented as means ± SEM. ‘n.s.’ indicates ‘not significant’.
(TIF)
