# Supplementary figures and images for: Macrolide Antibiotics Exhibit Cytotoxic Effect under Amino Acid-Depleted Culture Condition by Blocking Autophagy Flux in Head and Neck Squamous Cell Carcinoma Cell Lines
Source: PLoS One. 2016 Dec 15;11(12):e0164529. doi: 10.1371/journal.pone.0164529 (PMC5158196; doi:10.1371/journal.pone.0164529)

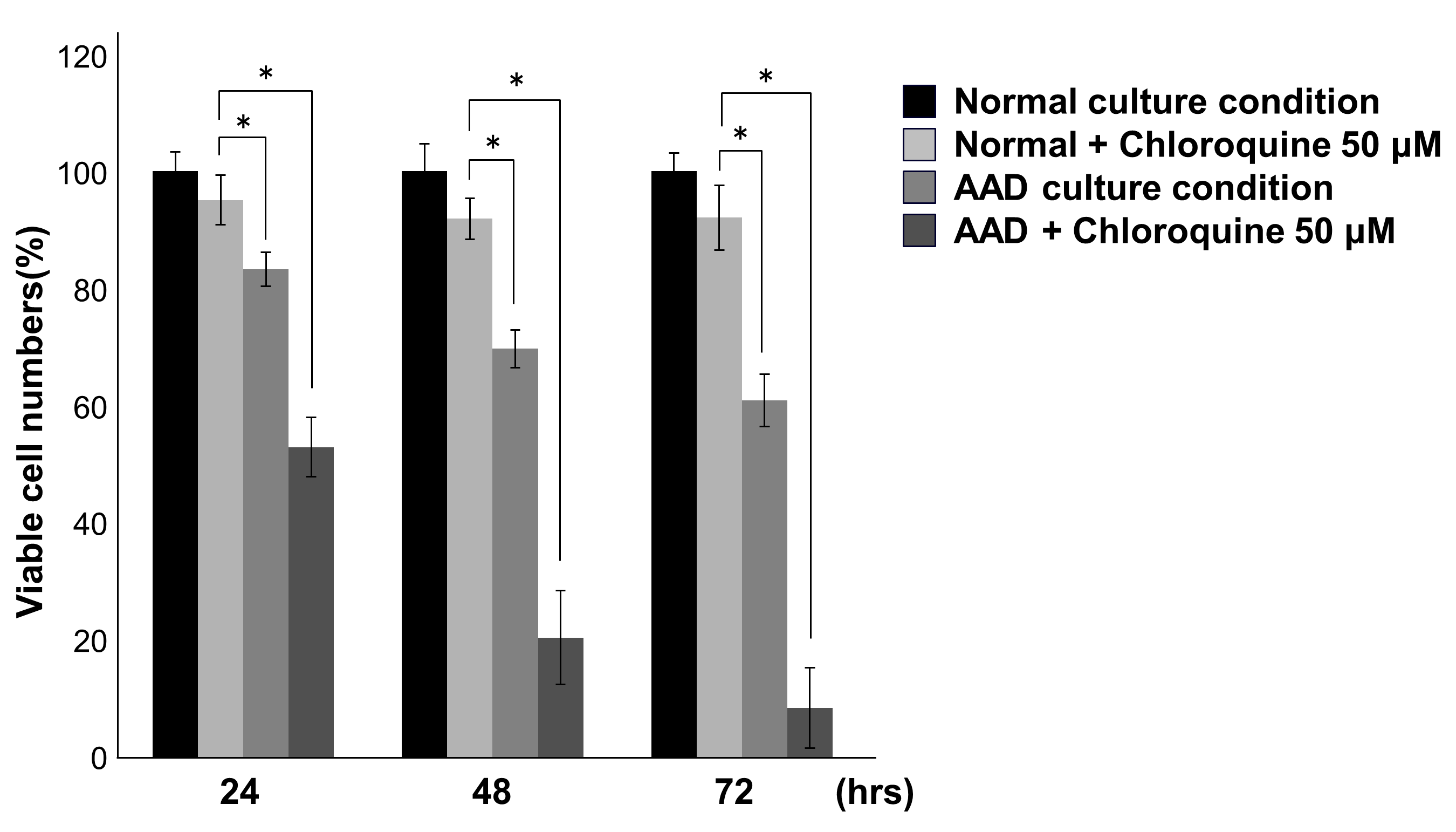

Supplement: S1 Fig — CAL 27 cells were cultured with/without 50 μM chloroquine either in the complete culture medium or AAD culture medium for the indicated period of time. Then, viable cell number was assessed and expressed as percentage to the viable cells cultured in the complete culture medium at each indicated culture period. Data are presented as means ± SEM. *p < 0.05. (TIF) [file pone.0164529.s001.TIF]

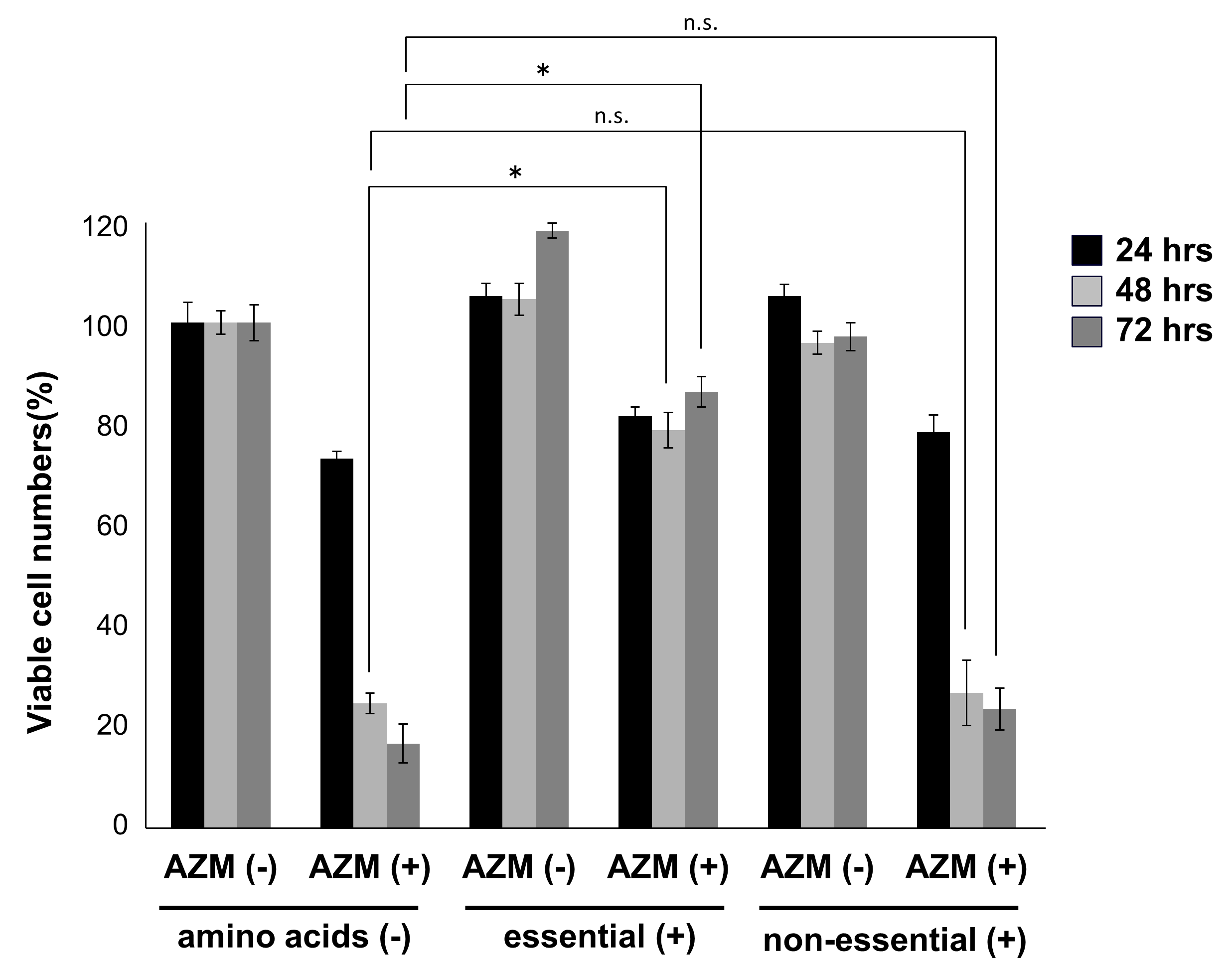

Supplement: S2 Fig — CAL 27 cells were cultured in the AAD culture medium supplemented with essential and non-essential amino acids with/without AZM (50 μM). ‘Essential’ and ‘non-essential’ indicate 2% MEM essential amino acids and 1% MEM non-essential amino acids at the final concentration (Wako), respectively. The number of viable cells was determined and compared with that of viable cells cultured under the AAD culture condition without AZM. *p < 0.05. ‘n.s.’ indicates ‘not significant’. (TIF) [file pone.0164529.s002.TIF]

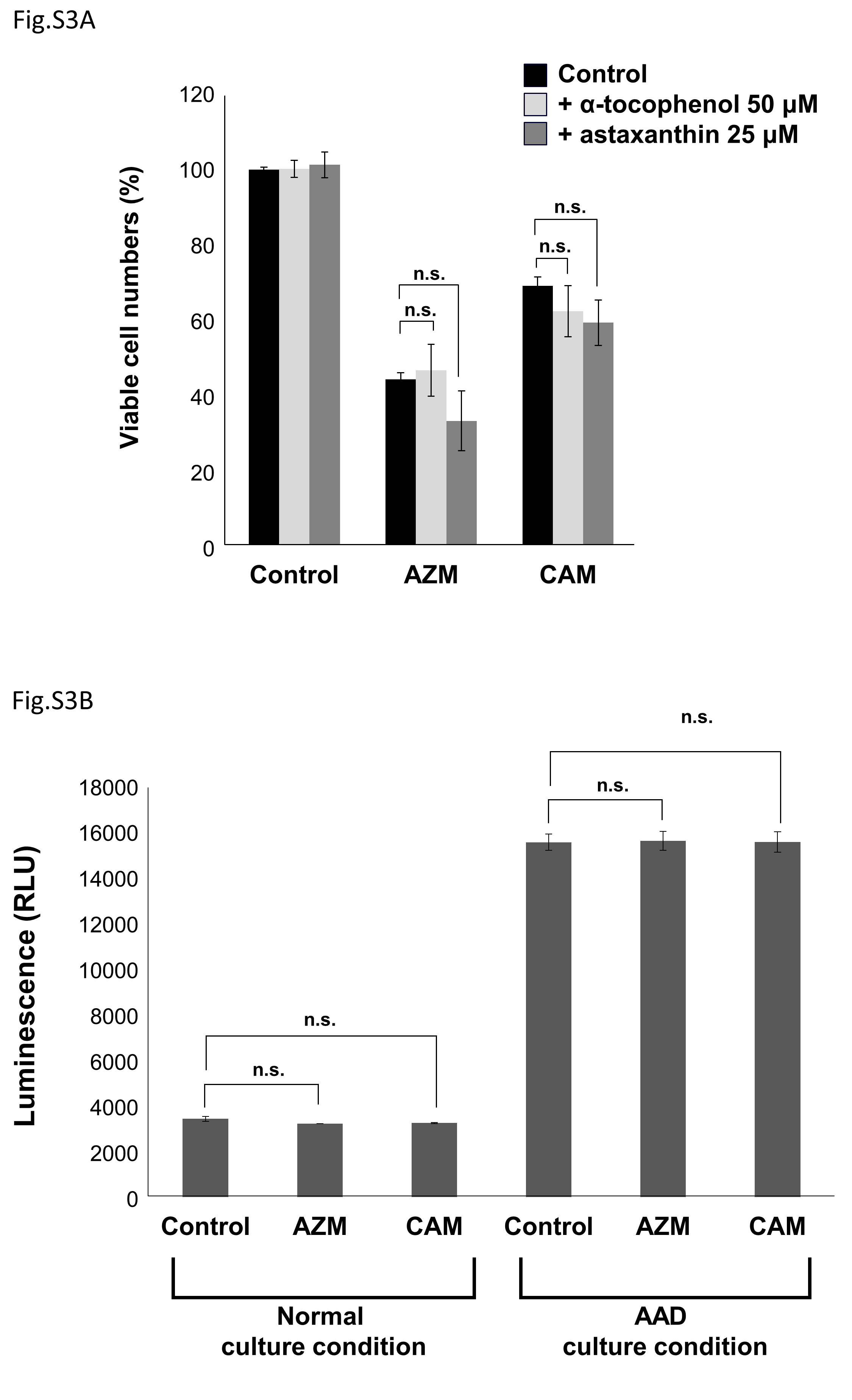

Supplement: S3 Fig — (A) CAL 27 cells were cultured with macrolides under the AAD culture condition with 10% FBS with/without the two types of ROS scavengers, namely, α-tocopherol (50 μM) and astaxanthin acid (25 μM) for 48 hrs. (B) CAL 27 cells were cultured with macrolides under the complete or AAD culture condition for 6 hrs. ROS production was assessed using ROS-Glo™ H2O2 Assay (Promega) as described in Materials and Methods. ‘n.s.’ indicates ‘not significant’. (TIF) [file pone.0164529.s003.tif]

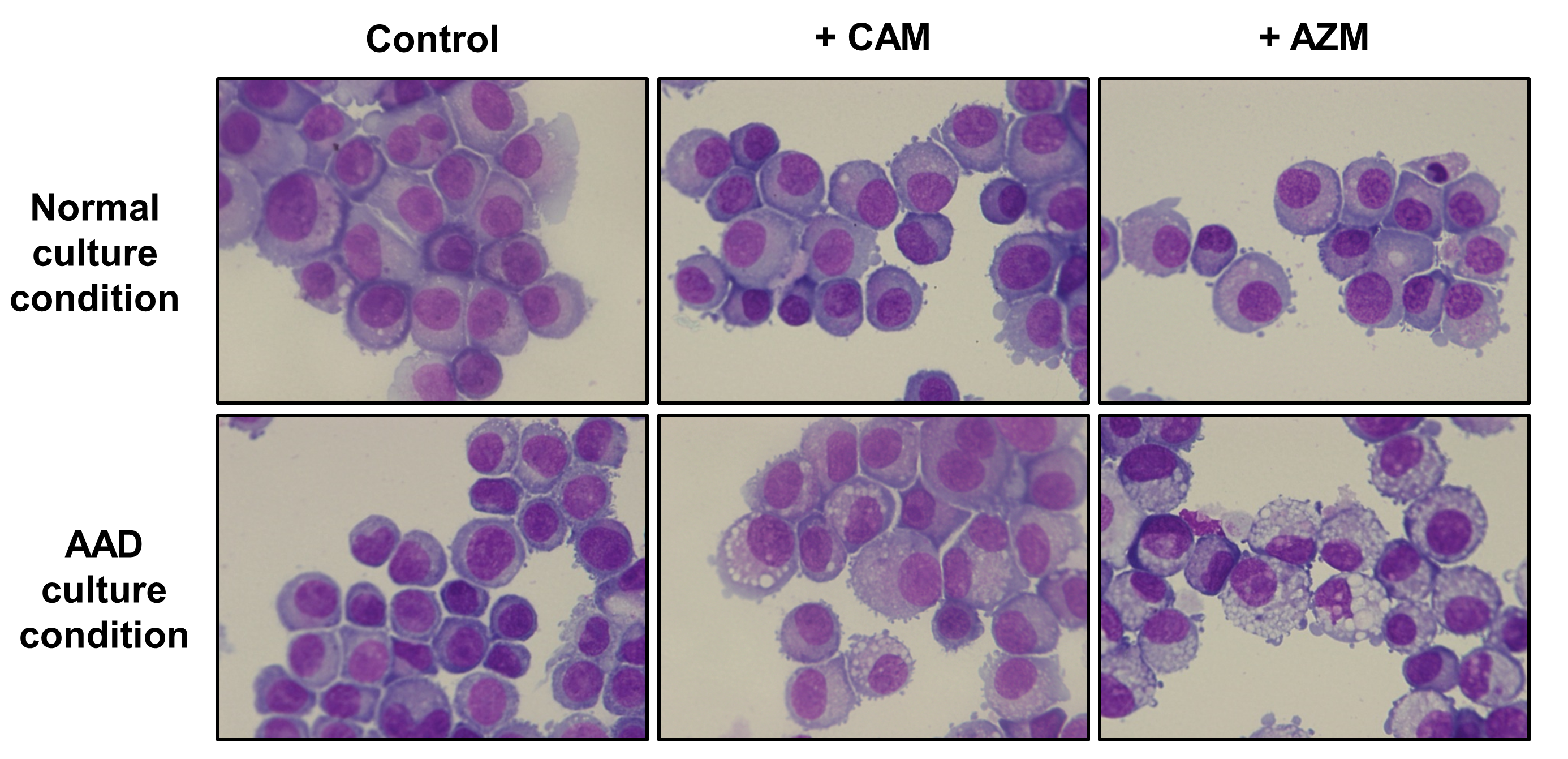

Supplement: S4 Fig — May-Grünwald-Giemsa staining was performed after treatment with or without macrolides under the normal or AAD culture condition for 24 hrs. (TIF) [file pone.0164529.s004.TIF]

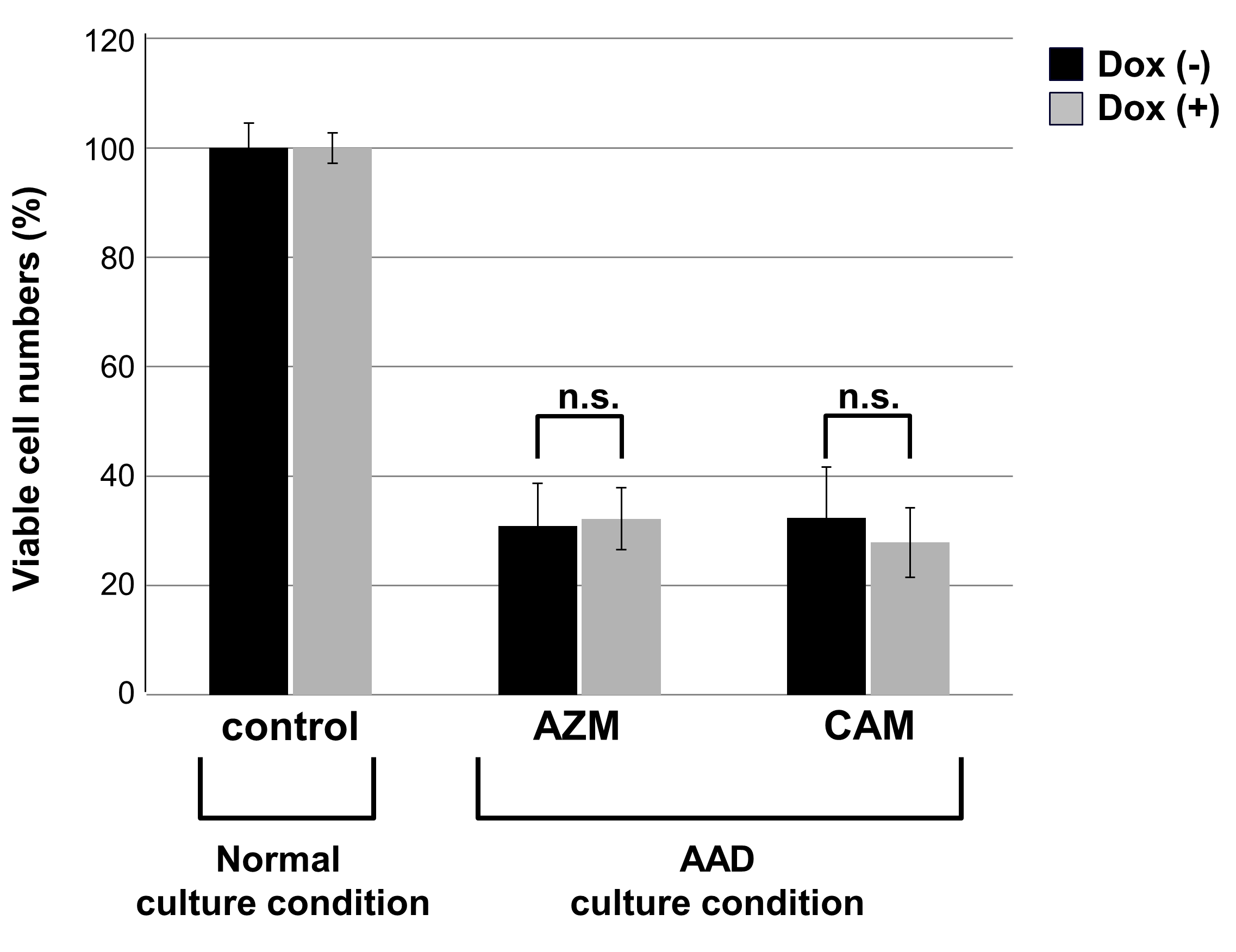

Supplement: S5 Fig — m5-7 cells with/without pretreatment with Dox (10 ng/mL) were cultured under the normal culture or AAD culture condition with AZM/CAM (50 μM) for 24 hrs. Viable cell number is expressed as the percentage of viable m5-7 cells with/without Dox under the normal culture condition. Data are presented as means ± SEM. ‘n.s.’ indicates ‘not significant’. (TIF) [file pone.0164529.s005.TIF]
